# Metformin attenuates the effect of *Staphylococcus aureus* on airway tight junctions by increasing PKCζ‐mediated phosphorylation of occludin

**DOI:** 10.1111/jcmm.13929

**Published:** 2018-11-18

**Authors:** Kameljit K. Kalsi, James P. Garnett, Wishwanath Patkee, Alexina Weekes, Mark E. Dockrell, Emma H. Baker, Deborah L. Baines

**Affiliations:** ^1^ Institute for Infection and Immunity St George's University of London London UK; ^2^ South West Thames Institute for Renal Research St Helier Hospital Carshalton UK; ^3^ Present address: Institute of Cellular Medicine Newcastle University Newcastle upon Tyne UK

**Keywords:** airway epithelium, metformin, occludin, PKCζ, respiratory infection, *Staphylococcus aureus*, tight junctions, ZO‐1

## Abstract

Airway epithelial tight junction (TJ) proteins form a resistive barrier to the external environment, however, during respiratory bacterial infection TJs become disrupted compromising barrier function. This promotes glucose flux/accumulation into the lumen which acts as a nutrient source for bacterial growth. Metformin used for the treatment of diabetes increases transepithelial resistance (TEER) and partially prevents the effect of bacteria but the mechanisms of action are unclear. We investigated the effect of metformin and *Staphylococcus aureus* on TJ proteins, zonula occludins (ZO)‐1 and occludin in human airway epithelial cells (H441). We also explored the role of AMP‐activated protein kinase (AMPK) and PKCζ in metformin‐induced effects. Pretreatment with metformin prevented the *S. aureus*‐induced changes in ZO‐1 and occludin. Metformin also promoted increased abundance of full length over smaller cleaved occludin proteins. The nonspecific PKC inhibitor staurosporine reduced TEER but did not prevent the effect of metformin indicating that the pathway may involve atypical PKC isoforms. Investigation of TJ reassembly after calcium depletion showed that metformin increased TEER more rapidly and promoted the abundance and localization of occludin at the TJ. These effects were inhibited by the AMPK inhibitor, compound C and the PKCζ pseudosubstrate inhibitor (PSI). Metformin increased phosphorylation of occludin and acetyl‐coA‐carboxylase but only the former was prevented by PSI. This study demonstrates that metformin improves TJ barrier function by promoting the abundance and assembly of full length occludin at the TJ and that this process involves phosphorylation of the protein via an AMPK‐PKCζ pathway.

## 1 INTRODUCTION

The airway epithelium of the human respiratory tract acts as the first physical barrier that protects against inhaled substances and pathogens. Epithelial barrier dysfunction during respiratory bacterial infections is characterized by alterations in tight junction (TJ) protein abundance.^1^, ^2^ TJs, the most apically located of the intercellular junctional complexes, regulate the passage of solutes, ions, and macromolecules through the paracellular space between epithelial cells. TJs are formed by three classes of integral transmembrane proteins: claudins,^3^ occludins,^4^ and junctional adhesion molecules; that bind to the actin cytoskeleton directly or through the intracellular scaffolding proteins zonula occludins (ZO).^5^ ZO‐1 is a cytoplasmic protein that plays an integral role in TJ organization by linking transmembrane components of TJs to the actin cytoskeleton.^6^

In the airway, TJs play an important role in restricting the movement of glucose from the interstitium into the airway surface liquid (ASL) limiting the availability of nutrients and contributing to defence against infection.^7^ Inflammatory stimuli such as bacterial infections induce changes in TJs which lead to increased permeability.^8^ Our group previously showed that treatment of H441 airway epithelial cell monolayers grown at air‐liquid interface with the biguanide metformin produced an increase in transepithelial electrical resistance (TEER). We showed that this was sufficient to attenuate *Staphylococcus aureus*‐induced increases in paracellular glucose movement across the epithelium, in turn leading to a reduction in apical glucose accumulation and diminished bacterial growth.^9^ This was also demonstrated in vivo where metformin reduced glucose flux across murine airways and a reduction in *S. aureus* growth.^9^

The effect of metformin on TEER likely reflects an alteration in TJ composition and/or assembly but the TJ proteins involved and the signalling pathways regulating such changes are unclear. We have shown that metformin activates AMP‐activated protein kinase (AMPK) in H441 cells^10^ and other AMPK agonists such as 5‐aminoimidazole‐4‐carboxamide‐1‐β‐d‐ribofuranoside (AICAR) 11 have also been reported to elevate TEER in other epithelial cells.^12^, ^13^, ^14^ Metformin‐induced increases in TEER in Calu3 cells were prevented with the AMPK antagonist Compound C,^13^ consistent with metformin increasing airway epithelium TEER through an AMPK‐dependent pathway.

It has been proposed that metformin‐induced increase in AMPK activity triggers the activation of extracellular related kinase (ERK), phosphoinositide dependent kinase 1, and atypical protein kinase C (aPKC) namely PKCζ and PKCλ/τ.^15^ How these proteins are involved in metformin‐induced increase in TEER has not been investigated in the airway, but evidence indicates that assembly and preservation of TJ involves phosphorylation with Ser/Thr kinases such as PKC.^16^ Occludin is highly phosphorylated on its Ser/Thr residues^17^ which can be readily dephosphorylated when the TJ are disrupted by calcium depletion, short exposure to phorbol esters, cholesterol depletion or bacterial infections.^2^, ^18^, ^19^, ^20^

The aims of this study were therefore (a) to investigate the effects of metformin on key airway epithelial TJ protein abundance; (b) to investigate whether metformin reversed the effects of *S. aureus* on TJs and (c) to identify downstream signalling molecules involved. Identifying the proteins involved in the protective effects of metformin on airway epithelial TJs during infection may lead to better understanding of how glucose permeates into the ASL and highlight potential therapeutic targets to prevent hyperglycaemia‐induced respiratory infections.

## 2 MATERIALS AND METHODS

### 2.1 Cell culture

Airway epithelial cells (H441) were grown on permeable membrane supports (Transwells, Corning, NY, USA) until confluent and then taken to air‐liquid interface to form polarized monolayers, as previously described.^21^ Monolayers were pretreated with 1 mM metformin (pharmacological dose to elicit changes in vitro) which was added to the basolateral medium for 18 hours before apical addition of *S. aureus*. A single colony of *S. aureus* strain 8325‐4 was selected and grown overnight in 37°C in RPMI media (Life Technologies, Paisley, UK) and diluted with glucose‐free RPMI to produce a culture of approximately 5 × 10^5^ CFU in 50 μL this was applied to the apical surface of H441 monolayer as described previously.^13^ Co‐cultures were placed in a CO_2_ incubator at 37°C for 7 hours, after which monolayers were immunostained for TJ or cell lysates were prepared for Western blot analysis. Transepithelial electrical resistance (TEER) was measured using a voltohmmeter (WPI, Hitchin, UK). H441 monolayers were pretreated with protein kinase inhibitors (50 nM staurosporine or 10 μM PKC pseudosubstrate or 80 μM Compound C were prepared in dimethyl sulfoxide) 30 minutes prior to the addition of metformin.

### 2.2 Immunofluorescence microscopy

Fully differentiated H441 at air‐liquid‐interface grown on clear transwell were initially fixed with methanol:acetone for 10 minutes at room temperature, washed with PBS. Cells were blocked and permeabilized with 1% bovine serum albumin containing 0.1% Triton made up in Tris‐buffered saline. Primary antibody for ZO‐1 was a rabbit polyclonal used at (1:100 dilution) from Thermofisher (61‐7300; Hemel Hempstead, UK). Occludin antibody was a rabbit polyclonal used at (1:100 dilution) from Santa Cruz Biotechnology (H‐279, Wembley, UK). Cells were incubated at room temperature for 1 hour washed with PBS and secondary antibody was added (anti‐rabbit Alexa Fluor 488 at 1:100 dilution) for 30 minutes at room temperature. After rinsing with PBS transwell membranes were cut with a clean scalpel and mounted onto slides with Vectashield mounting medium containing 4′,6‐diamino‐2‐phenylindole (DAPI) for nuclei staining. Images were visualized under a Zeiss LSM 510 Meta confocal fluorescence microscope (Cambridge, UK).

### 2.3 Western blot analysis for TJ proteins

Cell lysates were prepared by adding ERK phosphorylated buffer containing 20 mM Tris‐HCL pH 7.5, 150 mM NaCl, 1 mM EDTA, 50 mM NaF, 1 mM Na_3_VO_4_, 1% w/v Triton‐X, 0.5% w/v sodium deoxycholate, 0.1% w/v SDS, 1 μg/mL protease inhibitor cocktail (P8340; Sigma, Gillingham, UK). Cells were sonicated and debris was removed by centrifugation at 11 180 *g* at 4°C for 10 minutes. Total protein concentration was determined by Bradford Assay and between 30 and 60 μg was electrophoresed on NuPage Novex 10% Bis‐Tris Protein Gels (Invitrogen, Hemel Hempstead, UK). Protein was then transferred for 70 minutes onto polvinylidene difluoride membranes (Merck Millipore, Watford, UK). Blots were blocked in Odyssey Blocking Buffer for 1 hour at room temperature. Blots were incubated overnight with the following antibodies obtained from Life Technologies and used at 1:500 dilution; mouse anti E‐Cadherin (33‐4000), rabbit anti‐Claudin‐1 (51‐9000), and rabbit anti‐Occludin (H‐279; Santa Cruz Biotechnology). The blots were then washed in Tris‐buffered saline/0.2% Tween 20 and incubated with either Donkey antimouse IgG (926‐3221; Licor, Cambridge, UK) or goat anti‐rabbit IgG (925‐68071; Licor) for 1 hour at room temperature and kept in the dark. Blots were subsequently probed with a mouse monoclonal antibody to β‐actin. Detection of antigen‐antibody complexes was assessed using a Licor Odyssey Western blot imaging system. Integrated density of each fluorescent band was determined, using Image Studio Lite Ver 3.1 (Licor). Results are expressed as a ratio to β‐actin control within the same sample.

### 2.4 TJ assembly after calcium depletion

Polarized H441 cell monolayers grown on 12 well transwells were washed free of calcium with calcium free Kreb's solution containing 117 mM NaCl, 25 mM NaHCO_3_, 4.7 mM KCl, 1.2 mM MgSO_4_, 1.2 mM KH_2_PO_4_ and 5 mM glucose on both the apical and basolateral compartments. Low calcium Kreb's (calcium free Krebs with 2% Foetal calf serum, equivalent to 0.045 mM calcium from FCS) with 2 mM Ethylene‐bis(oxyethylenenitrilo)tetraacetic acid (EGTA)^22^ was added to both apical and basolateral compartments. TEER was measured over 20 minutes until this was reduced to ~50 Ω/cm^2^ from ~300 Ω/cm^2^. TJ re‐assembly was assessed by washing the cells free of EGTA and adding complete media containing normal calcium concentration (0.42 mM) and 2% FCS. The cells were allowed to recover for 24 hours, TJ integrity was assessed by measuring TEER and by immunofluorescence localization of occludin. Graphic profiles of occludin staining were created by analysing the distribution and intensity of pixels along a chosen line, using ImageJ software (ImageJ, Bethesda, MD, USA).

### 2.5 Immunoprecipitation

H441 cells treated for 18 hours with 1 mM Metformin ± 10 μM PKC pseudosubstrate were washed briefly with ice‐cold phosphate‐buffered saline and harvested into ice‐cold lysis buffer containing 20 mM Tris‐HCL pH 7.5, 150 mM NaCl, 1 mM EDTA, 50 mM NaF, 1 mM Na_3_VO_4_, 1% w/v Triton‐X, 0.5% w/v sodium deoxycholate, 0.1% w/v SDS, 1 μg/mL protease inhibitor cocktail (P8340; Sigma). Protein‐G Sepharose beads (Abcam, Cambridge, UK) were incubated for 2 hours with 5 μg anti‐occludin antibodies on an orbital shaker at 4°C. Cell lysates (1.0 mg protein/mL) were added to the coated beads and incubated overnight on an orbital shaker at 4°C. After several washes with PBS, protein bound to the beads was eluted by heating at 95°C for 5 minutes in Laemmli sample buffer, subjected to Western blot analysis, immunostained with anti‐phosphoserine (Thermo‐Scientific, Hemel Hempstead, UK) or anti‐phosphothreonine (Thermo‐Scientific) and visualized using ECL (BioRad, Watford, UK).

### 2.6 Statistical analysis

Data were analysed with Prism 4 (GraphPad, La Jolla, CA, USA) and ImageJ softwares. Data were obtained from at least two independent experiments and are represented as mean ± SEM. Statistical significance was evaluated, as indicated in figure legends, using unpaired Student's *t* test, one‐way ANOVA with post hoc Tukey test and nonparametric Kruskal‐Wallace and Mann‐Whitney test when sample size was less than 6. The alpha‐level used to determine significance was set at *P* < 0.05.

## 3 RESULTS

### 3.1 Metformin prevents *S. aureus*‐induced reduction in ZO‐1 and occludin expression at airway epithelial TJs

**Figure 1:**
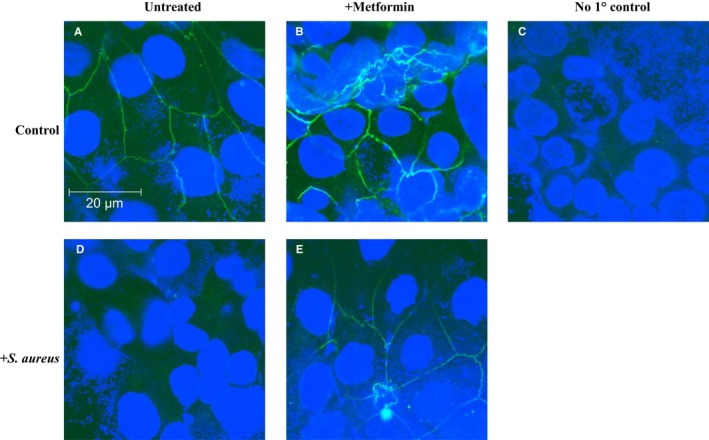
H441 polarized monolayers were fixed and stained for ZO‐1 expression in the presence and absence of *Staphylococcus aureus* co‐culture for 7 h. (A) Control cells no treatment. (B) Pretreatment with 1 mM metformin for 18 h. (C) In the absence of ZO‐1 antibody no Alexa Fluor 488 fluorescence was observed. (D) Co‐culture of H441 cells with *S. aureus* and cells pretreated with 1 mM metformin (E). The nuclei were counterstained with DAPI. All images were obtained at ×63 magnification. Bar = 20 μm

To examine the effects of metformin on proteins associated with TJs, changes in ZO‐1 localization at cellular junctions was determined by immunocytochemical analysis of H441 airway epithelial monolayers in the presence and absence of metformin pretreatment. Metformin treatment increased ZO‐1 expression at the TJ. Co‐culture with *S. aureus* led to loss of ZO‐1 at the TJ. Metformin pretreatment partially attenuated the effects of bacteria on ZO‐1 expression at the TJ (Figure 1A‐E).

**Figure 2:**
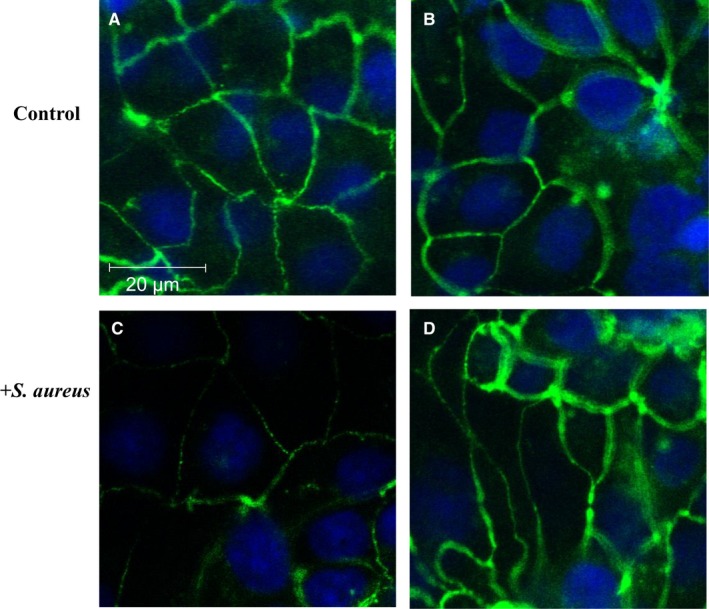
H441 polarized monolayers were fixed and stained for Occludin expression in the presence and absence of *Staphylococcus aureus* co‐culture for 7 h. (A) Control cells no treatment. (B) Pretreatment with 1 mM metformin for 18 h. (C) Co‐culture of H441 cells with *S. aureus* and cells pretreated with 1 mM metformin (D). The nuclei were counterstained with DAPI. All images were obtained at ×63 magnification. Bar = 20 μm

Occludin is a TJ‐associated transmembrane protein that binds to actin through its association with ZO‐1.^23^ Given that metformin increased ZO‐1 expression in H441 monolayers in the presence and absence of *S. aureus*, changes in occludin expression by immunocytochemistry were also investigated. Similar to ZO‐1 staining, co‐culture with *S. aureus* decreased occludin abundance at the cell junction. This was not observed when cells were pretreated with metformin (Figure 2A‐D).

### 3.2 Metformin increases occludin and attenuates *S. aureus*‐induced changes in abundance

**Figure 3:**
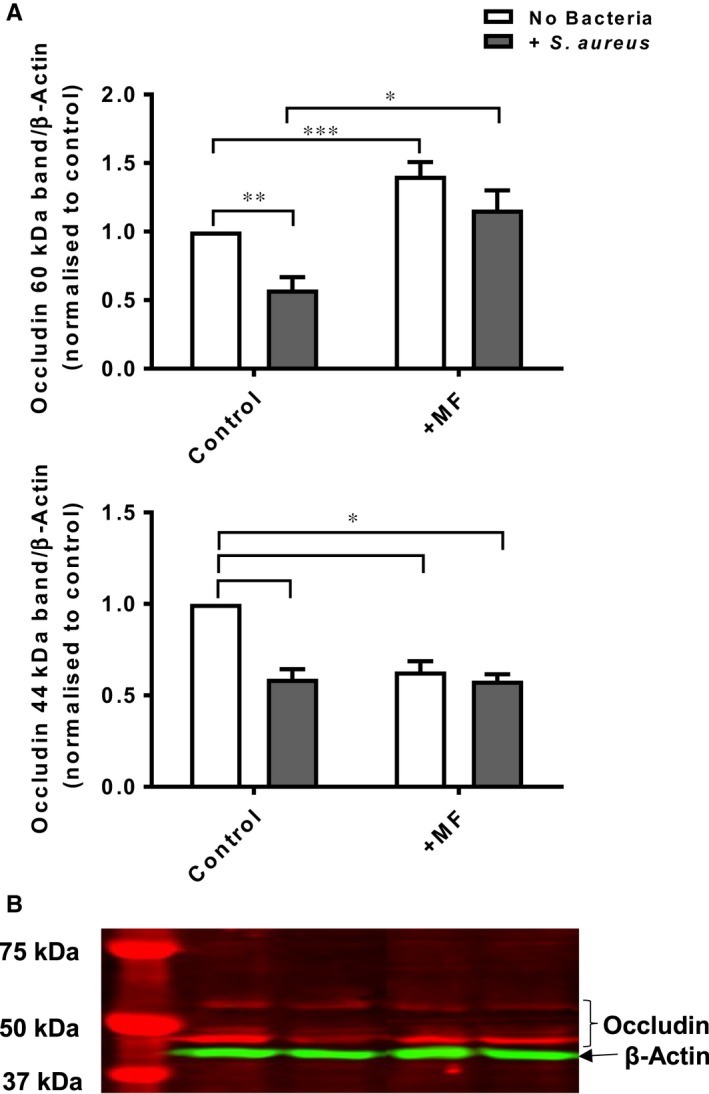
Metformin attenuates *Staphylococcus aureus* induced cleavage of occludin at 60 kD and alters cleavage fragments at 44 kD. (A) Occludin bands were normalized to β‐actin and to their respective controls,*P < 0.05, **P < 0.01, ****P < 0.0001 significantly different from control or H441 co‐culture with *S. aureus*, n = 4. (B) Western blot of H441 protein extracts of control, *S. aureus*, metformin/*S. aureus* immunoblotted for occludin and β‐actin

Metformin increased the abundance of the 60 kD protein (considered to be full length occludin) in H441 (Figure 3B) by 41 ± 10% (*P* < 0.001; n = 8). Co‐culture with *S. aureus* significantly reduced the abundance of the 60 kD protein by 34 ± 10% (*P* < 0.05; n = 7). This was prevented by pretreatment with metformin, which increased occludin abundance by 54 ± 12% compared to *S. aureus* (*P* < 0.05; n = 5) (Figure 3A). The 44 kD cleaved protein was also observed in our H441 lysates (Figure 3B). Both metformin and *S. aureus* significantly reduced the abundance of the 44 kD protein by 37 ± 5% and 41 ± 5% respectively (both *P* < 0.05; n = 4). Abundance was also lowered by 42 ± 3%, *P* < 0.05; n = 4, in cells pretreatment with metformin in the presence of *S. aureus* (Figure 3A). Taken together these data indicate that metformin promoted increased abundance of the full length occludin over the 44 kD cleavage product. Minor cleavage fragments at 46 and 38 kD were also observed (data not shown) but their abundance did not change significantly in the presence of *S. aureus*.

### 3.3 Metformin and co‐culture with *S. aureus* had no effect on Claudin‐1 abundance

**Figure 4:**
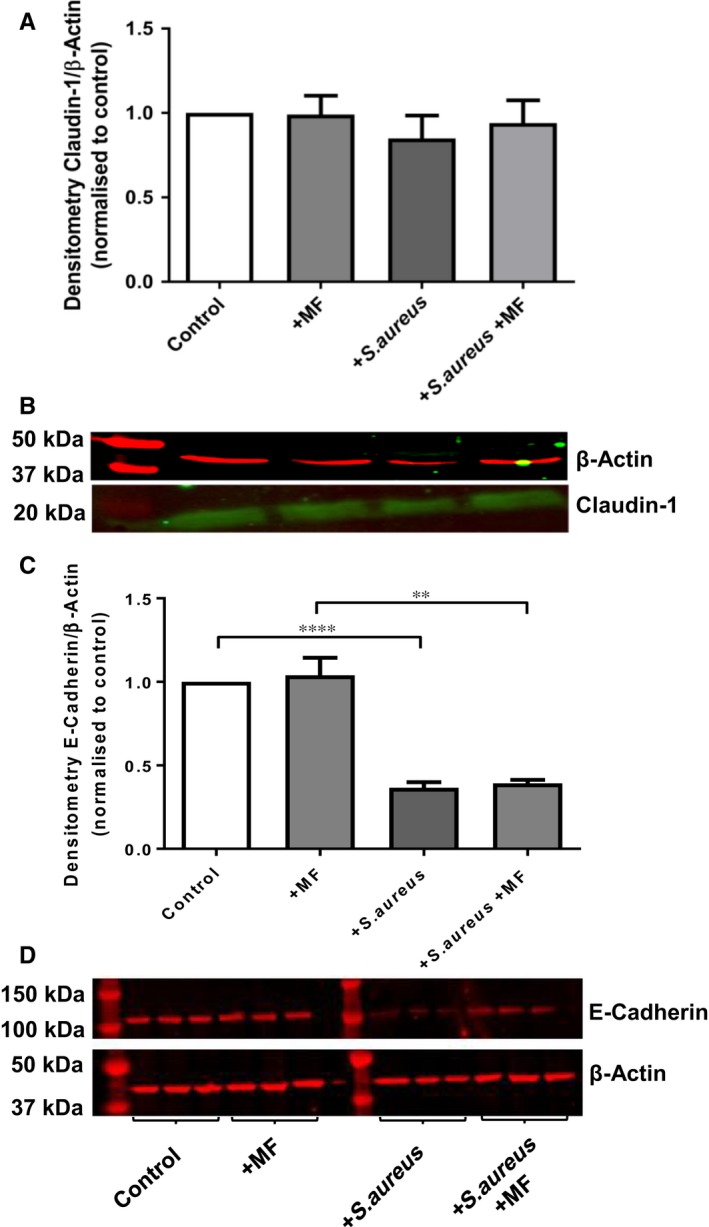
Differential effects of metformin and *Staphylococcus aureus* on Claudin‐1 and E‐cadherin abundance. (A and B) Claudin‐1 densitometry and immunoblot normalized to β‐actin and control, n = 3 showed no differences with treatment with 1 mM metformin or *S. aureus*. (C and D) E‐cadherin densitometry and immunoblot normalized to β‐actin and control. Co‐culture with *S. aureus* significantly reduced E‐cadherin abundance which was not prevented by the presence of metformin **P < 0.01, ***P < 0.001 ****P < 0.0001, n = 13 compared to control and metformin

Claudin proteins are considered to be the structural backbone of TJs^24^ and critical regulators of paracellular permeability.^25^ Changes in Claudin‐1 abundance, an integral transmembrane TJ protein expressed in airway epithelium, was therefore investigated. However, neither *S. aureus* addition nor metformin treatment had any effect on Claudin‐1 abundance in H441 monolayers (Figure 4A and B).

### 3.4 Metformin does not prevent the decrease in E‐cadherin abundance produced by co‐culture with *S. aureus*

In addition to occludin and claudin‐1, the cell adhesion molecule E‐cadherin was also examined, as disruption of E‐cadherin has been shown to prevent TJ formation.^26^, ^27^ *Staphylococcus aureus* addition significantly reduced E‐cadherin abundance by 63 ± 3% (*P* < 0.0001; n = 13) compared to control and metformin. However, metformin had no effect on the abundance of E‐cadherin in either the presence or absence of *S. aureus* (Figure 4C and D).

### 3.5 Metformin‐induced increase in TEER and occludin abundance is abrogated by PKCζ pseudosubstrate inhibitor

**Figure 5:**
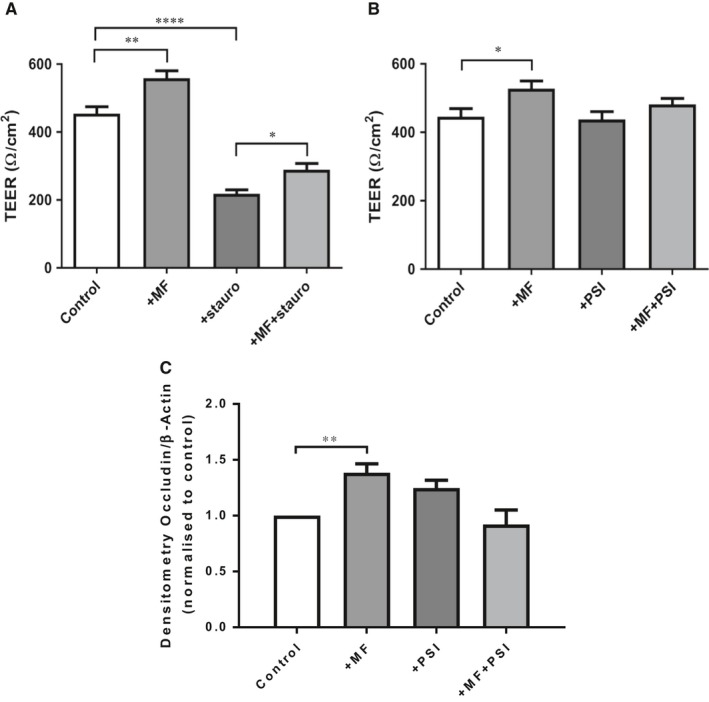
Staurosporine prevents metformin induced increase in transepithelial resistance (TEER). (A) H441 monolayers treated with 50 μM staurosporine in the presence or absence of 1 mM metformin significantly reduced TEER compared to control and metformin *P < 0.01, ****P < 0.0001, n = 4‐5. (B) In the presence of 10 μM PKC‐ζ pseudosubstrate inhibitor (PSI) TEER was attenuated **P < 0.01 compared to metformin, n = 6. (C) Densitometry of occludin in protein lysates extracted from H441 cells treated with PSI in the presence and absence of metformin. Occludin bands were normalized to β‐actin and to their respective controls, * **P < 0.01, compared to control, n = 4

To investigate the potential downstream targets of metformin that could be involved in the changes to TEER, we used the broad‐spectrum protein kinase inhibitor staurosporine. Addition of 50 nM staurosporine significantly reduced H441 TEER in the absence or presence of metformin from 454 ± 20 Ω/cm^2^ to 218 ± 12 Ω/cm^2^ (*P* < 0.0001; n = 5, compared to control) and from 559 ± 22 to 289 ± 19 Ω/cm^2^ (*P* < 0.0001; n = 4‐5), respectively, indicating a role for PKC isoforms in TJ formation and the generation of TEER. However, staurosporine did not prevent metformin elevation of TEER (*P* < 0.05, n = 5) (Figure 5A). Staurosporine inhibits several PKC isoforms at this concentration but much higher concentrations of staurosporine are required to inhibit PKCζ (1 μM). As staurosporine induces apoptosis at this concentration,^28^ we pretreated the epithelium with 10 μM PKCζ pseudosubstrate inhibitor (PSI) to test the effects of PKCζ inhibition on airway epithelial TEER. Treatment with PSI had no effect on H441 TEER. However, PSI prevented the metformin‐induced elevation of TEER (Figure 5B).

As the metformin‐induced changes in TEER were associated with increased occludin abundance, we further explored the role of PKCζ on occludin abundance. Treatment with PSI prevented the metformin‐induced increase in occludin abundance (Figure 5C) which reflects the TEER results observed in Figure 5B.

### 3.6 Metformin augments TJ reassembly and localization of occludin to the TJ after calcium depletion

**Figure 6:**
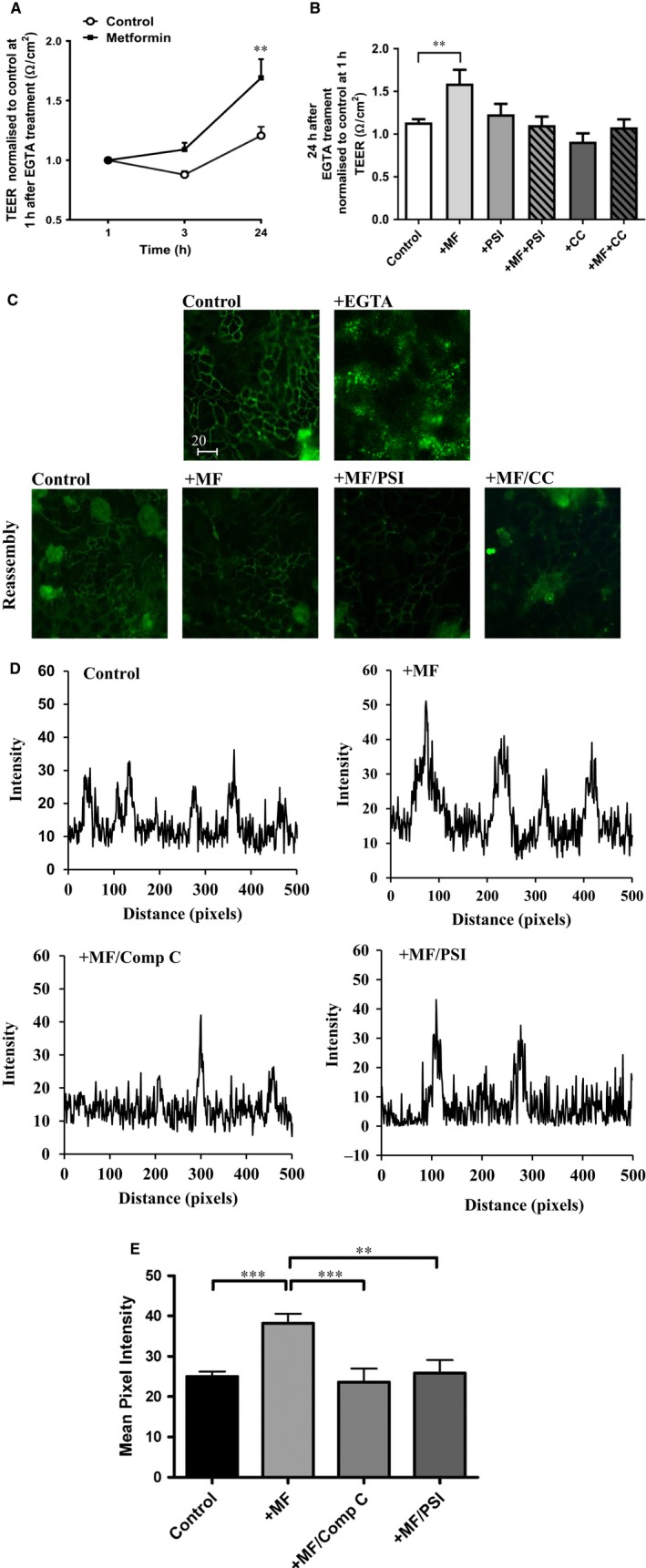
Metformin enhances TEER after calcium switch in H441 cells. (A) Time course of TJ re‐assembly after calcium replacement following depletion with 2 mM EGTA for 20 min in metformin treated and control monolayers. TEER was normalized to 1 h, **P < 0.01, n = 6 compared to control. (B) The effect of 10 μM PSI, 80 μM Compound C alone or combined with 1 mM metformin on TJ re‐assembly after calcium depletion. TEER was measured after 24 h and normalized to 1 h, **P < 0.01, n = 3‐6 compared to control. (C) Immunofluorescence of occludin staining before, 20 min after EGTA treatment and 24 h after EGTA was removed and replaced with regular medium containing calcium. Monolayers were pretreated with 1 mM metformin combined with 10 μM PSI or 80 μM Compound C staining shows the disruption of occludin after calcium depletion and reassembly 24 h later. All images were obtained at ×40 magnification. Bar = 20 μm. (D) Plots generated using ImageJ assess the intensity of occludin staining across cell junction reassembly after 24 h EGTA treatment. (E) Mean pixel intensity of metformin treated cells compared to control, ***P < 0.001, n = 3. Peak intensity was less in cells treated with metformin in the presence of PSI or Compound C, **P < 0.01 and ***P < 0.001, respectively, n = 3

To determine whether metformin aided the reassembly of TJs, cells were depleted of calcium to disrupt the TJ, calcium was then replaced and TEER was measured up to 24 hours later. Measurements were normalized to TEER after 1 hour recovery as a baseline to compare the reassembly of TJ. Metformin increased TEER and the rise in TEER was more rapid in metformin treated than in untreated control cells, *P* < 0.01, n = 8 (Figure 6A). Pretreatment of cells with the AMPK inhibitor, Compound C or PSI prevented the metformin‐accelerated restoration of TEER (Figure 6B).

Immunostaining of H441 cells 24 hours after calcium depletion was carried out to analyse the abundance, and localization of occludin at the TJs (Figure 6C). The intensity of occludin staining, was analysed for pixel intensity along a linear section taking in several cellular junctions (Figure 6D). The plots revealed an increase in peak intensity at the TJs in metformin treated cells 38.3 ± 2.3 compared to 25.0 ± 1.2 pixels in control, *P* < 0.001, n = 3. Peak intensity was less in cells treated with metformin in the presence of PSI or Compound C at 25.9 ± 3.2 and 23.6 ± 3.3 pixels (*P* < 0.01 and *P* < 0.001, respectively, n = 3) (Figures 6D and E).

### 3.7 Metformin increases Ser/Thr phosphorylation of occludin

**Figure 7:**
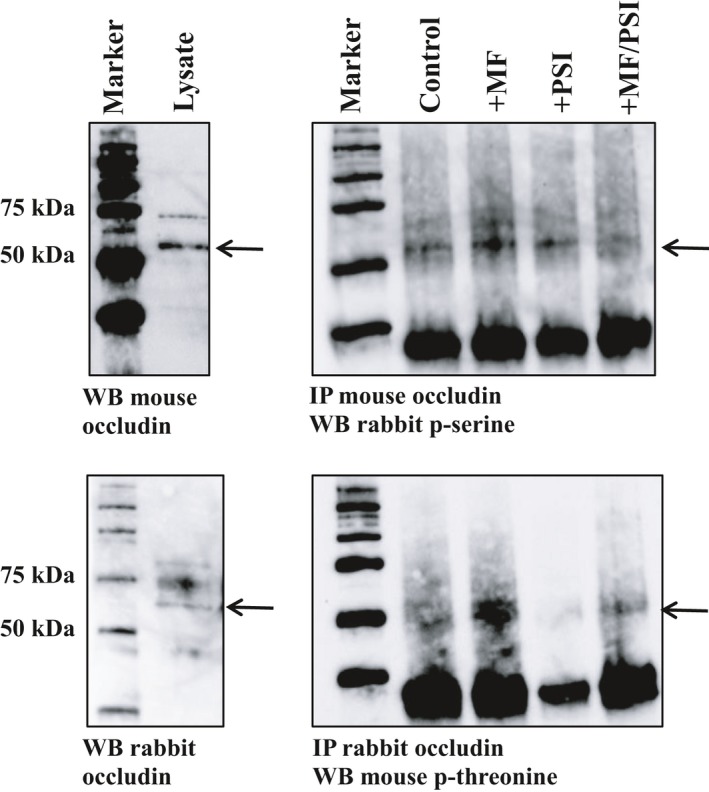
Occludin was immunoprecipitated from treated H441 cells. Monolayers were pretreated with 10 μM PSI alone or combined with 1 mM metformin. Immunocomplexes were then immunoblotted with phosphoserine and phosphothreonine. A nonimmunoprecipitated control cell lysate shows occludin bands at ~60 kD and ~44 kD

To determine the effect of metformin on Ser and Thr phosphorylation of occludin, protein extracts were immunoprecipitated with occludin and immunoblotted with p‐Ser and p‐Thr. Metformin treatment increased Ser/Thr phosphorylation of occludin compared to control. PSI reduced the metformin‐induced Ser/Thr phosphorylation of occludin to levels observed in controls (Figure 7). Treatment with PSI did not inhibit AMPK‐mediated phosphorylation of acetyl co carboxylase (ACC) (phospho‐ACC/total ACC was 4.2 ± 1.3 vs 4.7 ± 1.7 in metformin vs metformin/PSI treated cells, *P *=* *0.40, n = 4).

## 4 DISCUSSION

Our data indicates that metformin increases airway epithelial TEER by a process that involves increased ZO‐1 expression and increased abundance and assembly of full length occludin at the TJ. Importantly metformin produced a similar effect on ZO‐1 and occludin even in the presence of *S. aureus*, indicating that these proteins may be key TJ targets for metformin action and its ability to mitigate the effects of *S. aureus* on epithelial permeability.^13^

Epithelial barrier function is critical for host defence. It is important for maintaining the composition and innate immune function of the ASL and preventing access of pathogens to the internal mileu. TJs are key to barrier function of the epithelium and comprise a number of proteins which control the permeability of the epithelium to ions and solutes such as glucose.^29^, ^30^, ^31^ It has been shown that occludin spans the membrane four times with two extracellular loops and that ZO‐1 interacts with the C‐terminal domain which is essential for TJ assembly.^4^ Pathogenic microorganisms such as *S. aureus* have coat constituents such as lipoteichoic acid and express proteins on their surfaces such as Protein A which interact with epithelial cell surface receptors. They also release factors such as the pore forming α‐toxin which initiate host defence mechanisms but are toxic. These factors cause changes to epithelial cell function and can lead to apoptosis and necrosis if bacterial load increases.^32^ In our study, we found that the presence of *S. aureus* caused a reduction in ZO‐1, E‐cadherin and occludin. These findings are consistent with previous studies investigating the effects of *S. aureus* α‐toxin on human intestinal epithelial cells^33^ and protein A on airway epithelial cells.^34^ In contrast, metformin increased ZO‐1 and occludin and attenuated the reduction caused by *S. aureus*. These findings are similar to those from secretory airway Calu‐3 cells infected with *Pseudomonas aeruginosa* 14 suggesting that this is a common pathway in airway epithelial cells, affected by both gram +ve and −ve pathogens.

We observed that occludin in H441 cells presented as a protein of ~60 kD, with cleavage products of 46, 44 and 38 kD which were similar to that reported for Calu3 epithelial cells. A diverse range of proteases are known to cleave junctional proteins, calpains and elastases have been shown to cleave occludin.^35^, ^36^ There was a reciprocal relationship between the 60 and 44 kD occludin proteins before and after treatment with metformin indicating that metformin promoted increased abundance of full length over cleaved occludin. *Staphylococcus aureus* reduced abundance of both 60 and 44 kD products but in the presence of metformin the full length product was more abundant. Thus, we propose that metformin inhibited cleavage of occludin although the process by which this occurs requires further investigation.

Our data also indicate that metformin accelerated reassembly of TJs after calcium depletion, as measured by the elevation in TEER, and increased localization and abundance of occludin at the TJ. Thus, we propose that increased abundance of full length occludin and its assembly at the TJ is a critical determinant of the metformin‐induced elevation of TEER. Unlike Calu3 cells treated with *P. aeruginosa*,^14^ claudin‐1 abundance was not changed in H441 cells treated with either *S. aureus* or metformin which may reflect differences in cell type and/or pathogen. Other claudins were not investigated.

Metformin is known to activate AMPK in H441 cells^10^ and other activators of AMPK such as AICAR have been shown to increase TEER in airway cells,^14^ maintain cell polarity and barrier function.^37^, ^38^ We showed that Compound C prevented the metformin‐induced rise in TEER, TJ re‐assembly, and the increased abundance and localization of occludin at the TJ. Although, Compound C is known to inhibit a number of kinases in addition to AMPK,^39^, ^40^ these data support a role for AMPK in the signalling pathway. We found that staurosporine reduced basal TEER but did not prevent the metformin‐induced rise in TEER. At 50 nM, staurosporine inhibits a number of kinases, including PKC (IC50 = 3 nM),^41^, ^42^ protein kinase A (IC50 = 7 nM), p60v‐src tyrosine protein kinase (IC50 = 6 nM) and CaM kinase II (IC50 = 20 nM). In addition, staurosporine inhibits many of the conventional and novel isoforms of PKC, including PKCα (IC50 = 2 nM), PKCγ (5 nM), PKCη (4 nM), PKCδ (20 nM) and PKCε (73 nM). Thus, any of these kinases could be important for TJ formation but not metformin‐induced effects. Staurosporine does not inhibit PKCζ at this concentration but inhibition of PKCζ with a pseudosubstrate prevented the metformin‐induced increase in TEER, rise in occludin abundance and assembly at TJs.

We found that PSI did not influence phosphorylation of the target of AMPK ACC which would indicate that PKCζ acts downstream of AMPK. Phosphorylation of liver kinase B1 by PKCζ was shown to be necessary for metformin activation of AMPK in A549 distal airway cells.^43^ However, our data is supported by evidence from heart cells where metformin increased phosphorylation of PKCζ and that PKCζ activity did not influence metformin phosphorylation of AMPK.^44^

Finally, we show that metformin increased phosphorylation of occludin via PKCζ. We propose that this mechanism drives TJ assembly and the resultant rise in TEER. Inhibition of phosphorylation on a number of Thr‐residues in the carboxyterminus of the protein delayed assembly of full length occludin to the TJ and disrupted barrier function.^17^, ^18^

In summary, these data indicate a metformin‐AMPK‐PKCζ dependent pathway that maintains the airway epithelial barrier and assembly of TJs in the face of disruption by pathogens such as *S. aureus*. Our results indicate that occludin is a key target protein involved in this process. We cannot rule out that other additional metformin‐AMPK‐mediated effects, such as phosphorylation of G‐alpha vesicle associated protein (GIV), also contribute to TJ formation.^45^ Nevertheless, as we previously showed that metformin decreased glucose permeability across the airway epithelium, these data also implicate occludin as an important regulator of solute (glucose) permeability across airway epithelial TJs. Indeed, occludin has been linked to the regulation of paracellular diffusion of small molecules.^46^ Thus occludin and its phosphorylation by PKCζ may represent therapeutic targets for the prevention of hyperglycaemia‐associated respiratory infections by restricting glucose flux and accumulation in the ASL and thereby limiting the availability of glucose as a growth substrate for bacteria.

## CONFLICT OF INTEREST

The authors confirm that there are no conflicts of interest.

## AUTHOR CONTRIBUTION

DLB, EHB, JPG and KKK designed the research study; WP, AW, JPG and KKK performed the research; WP, AW, MED, DLB, JPG and KKK analysed the data; DLB, JPG and KKK wrote the paper.
